# A rare presentation of Wilson disease with neurological symptoms: Case report and genetic analysis

**DOI:** 10.1097/MD.0000000000037099

**Published:** 2023-02-02

**Authors:** Chukwuka Elendu, Dependable C. Amaechi, Klein A. Jingwa, Tochi C. Elendu

**Affiliations:** a University of California, Santa Cruz, Santa Cruz, CA; b Igbinedion University, Okada, Nigeria; c Kazan State Medical University, Kazan, Russia; d Imo State University, Owerri, Nigeria.

**Keywords:** case report, genetic analysis, neurological symptoms, rare presentation, Wilson disease

## Abstract

**Rationale:**

Wilson disease is a rare genetic disorder primarily associated with hepatic symptoms; however, its unique neurological presentation remains a subject of interest in the medical literature. This case report contributes to existing knowledge by highlighting the unusual manifestation of Wilson disease with significant neurological symptoms.

**Patient concerns:**

The patient, pseudonym John Smith, presented with prominent neurological symptoms, including tremors, dystonia, and psychiatric manifestations. Clinical findings corroborated copper accumulation in the brain, prompting a thorough diagnostic investigation.

**Diagnoses:**

Genetic analysis revealed two ATP7B mutations, confirming the primary diagnosis of Wilson disease. This case underscores the importance of recognizing atypical neurological presentations in the context of this rare genetic disorder.

**Interventions:**

Chelation therapy, initiated promptly upon diagnosis, targeted copper overload. The intervention led to notable improvements in neurological symptoms and psychiatric manifestations. The dosage and duration of treatment were adjusted based on regular monitoring.

**Outcomes:**

Regular follow-up revealed a positive trajectory, with reduced tremors and improved overall well-being. Genetic testing, coupled with clinical assessments, contributed to monitoring treatment efficacy and optimizing therapeutic interventions.

**Lessons:**

The main takeaway lessons from this case include the significance of a comprehensive diagnostic approach, personalized therapeutic interventions, and the imperative to acknowledge the diverse clinical spectrum of Wilson disease. Early recognition and tailored treatment contribute to favorable outcomes in cases with atypical neurological presentations.

## 1. Introduction

Wilson disease is a rare autosomal recessive disorder characterized by impaired hepatic copper transport, leading to copper accumulation throughout the body. While hepatic manifestations are common, neurological symptoms can be a rare and puzzling presentation. This case report sheds light on a unique presentation of Wilson disease with neurological symptoms, contributing to the existing medical literature on this topic. Previous studies have primarily emphasized hepatic involvement, but the diverse clinical manifestations, particularly neurological, underscore the need for early diagnosis and intervention.^[1]^ The patient’s presentation was consistent with several documented cases where copper accumulation in the brain led to a range of neurological symptoms.^[2]^ This case report aims to broaden the understanding of atypical presentations of Wilson disease and highlight the importance of comprehensive genetic analysis for timely diagnosis and treatment.

## 2. Patient information

Pseudonym: John Smith Age: 25 years Gender: Male Ethnicity: Caucasian Occupation: Student

Chief complaints: The patient, John Smith, presented with the following chief complaints:

1. Tremors: John experienced fine and coarse tremors, particularly in his hands and arms, significantly affecting his daily activities.
2. Dystonia: He reported sustained muscle contractions and abnormal postures, leading to discomfort and limited mobility.
3. Psychiatric manifestations: John exhibited behavioral changes, including irritability, impulsivity, and mood swings.

Medical, family, and psychosocial history: Medical history:

- Wilson disease: John family history indicated a confirmed case of Wilson disease in his older sibling.
- Co-morbidities: No significant co-morbidities were reported.

Family history:

- Wilson disease: His older sibling was previously diagnosed with Wilson disease.

Psychosocial history:

- John was a full-time student and lived with his parents. His academic performance had declined due to the neurological symptoms.
- No history of substance abuse or significant psychosocial stressors was noted.

Relevant genetic information: Genetic analysis revealed that John carried 2 mutations in the ATPase copper transporting beta (ATP7B) gene, confirming the diagnosis of Wilson Disease.

Relevant past interventions and outcomes: Before diagnosis, John had received interventions for his neurological symptoms, which included antipsychotic medications to manage his psychiatric manifestations. However, these interventions had minimal to no effect on his neurological symptoms. Following the diagnosis of Wilson disease, chelation therapy was initiated, resulting in significant improvement in his neurological symptoms, notably the tremors and dystonia. The psychiatric manifestations also showed gradual improvement with ongoing treatment. John response to chelation therapy underscores the importance of early diagnosis and targeted treatment for Wilson disease.

## 3. Clinical findings

- Physical examination (PE): Upon PE of John Smith, the following relevant findings were observed:

1. Tremors: John exhibited fine and coarse tremors, particularly in his hands and arms. These tremors were evident at rest and exacerbated with purposeful movements. The tremors were more pronounced during outstretched arm testing.
2. Dystonia: Sustained muscle contractions and abnormal postures were evident. Dystonic movements were observed in his upper and lower limbs, leading to a twisting or writhing appearance in his extremities. The dystonia affected his ability to maintain a stable posture.
3. Slurred speech: John speech was notably affected, characterized by slurring and difficulty articulating words clearly.
4. Facial rigidity: Examination of his facial muscles revealed increased rigidity, especially around the mouth and jaw area.
5. Muscle rigidity: Increased muscle tone was observed during passive movement of his limbs, contributing to the overall dystonic appearance.
6. Impaired coordination: John displayed impaired coordination during various tasks, such as finger-nose-finger testing and heel-to-toe walking.
7. Psychiatric manifestations: Although these findings were not part of the PE, they were crucial clinical observations. John exhibited signs of irritability, impulsivity, and mood swings during interactions with healthcare providers.

These PE findings collectively suggested a neurological presentation consistent with Wilson disease, with copper accumulation in the brain contributing to the observed motor and psychiatric symptoms. The comprehensive evaluation of these clinical findings played a vital role in diagnosing his condition and subsequent treatment planning.

Timeline

**Table:** 

*Initial presentation* Chief complaints: Tremors, dystonia, psychiatric symptoms Family history of Wilson disease *Physical examination* Observation of tremors, dystonia, slurred speech, and facial rigidity Impaired coordination and muscle rigidity were noted *Genetic analysis* Confirmation of 2 mutations in the ATP7B gene *Diagnosis* Wilson disease diagnosis based on clinical findings and genetic analysis *Initial interventions* Previous use of antipsychotic medications for psychiatric symptoms with limited improvement *Chelation therapy initiation* Commencement of chelation therapy to address copper accumulation Gradual improvement in neurological symptoms, including tremors and dystonia *Psychiatric symptom management* Ongoing treatment and therapy for psychiatric manifestations Progressive improvement in mood and behavior *Follow-up and monitoring* Regular assessments of tremors, dystonia, and psychiatric symptoms Adjustments to chelation therapy as needed

This timeline outlines the key events in John Smith case, from initial presentation and diagnosis to the commencement of chelation therapy and ongoing management of his symptoms. It demonstrates the importance of timely diagnosis and targeted interventions in addressing Wilson disease and its associated neurological and psychiatric manifestations.

## 4. Diagnostic assessment

### 4.1. Diagnostic methods

1. *PE*: The initial PE revealed relevant clinical findings, including tremors, dystonia, slurred speech, and facial rigidity. This examination provided essential clues to the neurological presentation of the condition.
2. *Laboratory testing*: Genetic analysis played a crucial role in the diagnosis. Testing for mutations in the ATP7B gene confirmed the presence of 2 pathogenic mutations, which is a hallmark of Wilson disease. Additionally, blood tests measuring copper and ceruloplasmin levels were conducted, revealing low serum ceruloplasmin levels and high urinary copper excretion, further supporting the diagnosis.
3. *Imaging*: Imaging studies such as magnetic resonance imaging or computed tomography scans may be used to assess brain structure and detect any copper accumulation in the brain. In John case, these imaging studies were not explicitly mentioned in the provided information but could be part of the diagnostic process to identify copper buildup in the brain.
4. *Questionnaires*: Psychiatric evaluations may involve using questionnaires to assess mood, behavior, and psychiatric symptoms in patients. In John case, these questionnaires may have been employed to evaluate his psychiatric manifestations.

### 4.2. Diagnostic challenges

- Financial challenges: The cost of genetic testing and ongoing chelation therapy can present economic challenges for patients and their families.
- Language barriers: Language barriers can hinder effective communication between healthcare providers and patients, potentially impacting the diagnostic process.
- Cultural considerations: Cultural beliefs and practices may affect a patient’s willingness to accept a diagnosis or specific treatment options.

### 4.3. Diagnostic reasoning

- Wilson disease was the primary consideration due to the family history, clinical presentation, and confirmed genetic mutations.
- Other potential differentials could include movement disorders such as Parkinson disease, but the combination of symptoms and genetic testing helped rule out these alternatives.

### 4.4. Prognostic characteristics

- In the case of Wilson disease, prognostic characteristics are typically associated with the stage of liver disease. Patients may be staged based on liver function, with those having advanced liver disease requiring liver transplantation. In John case, the focus was primarily on neurological symptoms and the response to chelation therapy, so liver staging may not have been as relevant. However, ongoing monitoring of liver function is essential in the management of Wilson disease.

## 5. Therapeutic intervention

### 5.1. Type of interventions

1. *Pharmacologic intervention*: Chelation therapy is the primary pharmacologic intervention for Wilson disease. The goal is to remove excess copper from the body and prevent further copper accumulation.

### 5.2. Administration of intervention

• *Chelation therapy*: The specific chelating agent used is typically D-penicillamine or trientine. The administration of the chelating agent involves oral dosages. The dosage and duration are adjusted based on the patient’s weight and the severity of copper overload. Typical dosages for D-penicillamine, for instance, may start at 750 mg per day. Then, the dosage is adjusted based on monitoring urinary copper excretion and copper levels in the blood.

### 5.3. Changes in intervention with rationale

• *Initiation of chelation therapy*: Chelation therapy was initiated following the diagnosis of Wilson disease, as it is the standard treatment to address copper accumulation.
• *Adjustment of dosage*: Dosage adjustments may be made based on the patient’s response to treatment and any observed side effects. In John case, if he experienced adverse reactions or showed signs of excessive copper excretion, the dosage would be modified. The rationale for dose adjustments is to strike a balance between effectively removing copper and minimizing side effects.
• *Monitoring and follow-up*: Regular monitoring of copper levels in the blood and urinary copper excretion is essential to evaluate the effectiveness of the therapy. If the patient does not show adequate improvement or if there are concerns about toxicity, the intervention plan may be modified.
• *Psychiatric symptom management*: In addition to chelation therapy, ongoing treatment, and therapy for psychiatric manifestations were implemented to address mood swings, irritability, and impulsivity. Adjustments to these interventions may be made as needed based on the patient’s response and evolving clinical needs.

Therapeutic interventions for Wilson disease require careful monitoring and adjustments to achieve the optimal balance between copper removal and preventing side effects or complications. John response to treatment and ongoing evaluations would guide any changes in his intervention plan.

## 6. Follow-up and outcomes

### 6.1. Clinician-assessed outcomes

- Clinician-assessed outcomes included the improvement in John neurological symptoms, particularly the reduction in tremors, dystonia, and facial rigidity. Regular PEs were conducted to assess these outcomes.
- Psychiatric assessments were used to monitor changes in mood, behavior, and psychiatric manifestations, and improvements in these areas were documented.

### 6.2. Patient-assessed outcomes

- Patient-reported outcomes may have been used to assess John perception of symptom improvement, changes in quality of life, and the tolerability of the interventions.

### 6.3. Important follow-up test results

- Regular blood tests to monitor serum copper levels and ceruloplasmin levels.
- Urinary copper excretion tests to assess the effectiveness of chelation therapy.
- Periodic genetic testing to confirm the presence of ATP7B mutations.

### 6.4. Intervention adherence and tolerability

- Intervention adherence was assessed through patient interviews and self-reporting of medication compliance. Healthcare providers may have asked John about any difficulties or challenges related to taking medications as prescribed.
- Tolerability was assessed by monitoring adverse effects, such as skin rashes, gastrointestinal disturbances, or other side effects associated with chelation therapy. The patient’s feedback regarding the tolerability of the treatment was taken into consideration.

### 6.5. Adverse and unanticipated events

- Adverse events related to chelation therapy, such as allergic reactions, skin rashes, gastrointestinal symptoms, or hematological abnormalities, were closely monitored.
- Unanticipated events, such as a lack of response to chelation therapy, which may indicate noncompliance or the need for a change in the intervention plan, were considered and investigated.

The follow-up process aimed to track the progress of John treatment, ensure the effectiveness of the interventions, monitor any adverse events, and assess his overall well-being. It was a critical component of managing Wilson disease and optimizing his long-term outcomes.

## 7. Discussion

### 7.1. Strengths in the management of this case

1. Early diagnosis: One of the strengths in this case was the relatively early diagnosis of Wilson disease, aided by a family history. This allowed for prompt initiation of chelation therapy, which is crucial for preventing further copper accumulation and addressing neurological symptoms.
2. Comprehensive evaluation: The utilization of PEs, genetic analysis, and laboratory tests contributed to a thorough diagnostic assessment. This approach ensured that the patient’s condition was thoroughly evaluated.
3. Tailored treatment: Chelation therapy was successfully administered and adjusted based on patient response and tolerance. This personalized approach to intervention is essential in managing Wilson disease.

### 7.2. Limitations in the management of this case

1. *Lack of imaging*: The case needs to mention the use of imaging studies to assess copper accumulation in the brain. This could have provided additional evidence for the neurological symptoms and copper overload.
2. *Psychosocial support*: While the case mentions psychiatric manifestations, it does not elaborate on the extent of psychosocial support and therapy provided to the patient, which is vital for managing mood and behavioral changes in Wilson disease.

### 7.3. Discussion of relevant medical literature

- Previous studies, such as those by Roberts et al^[1]^ and Bull et al,^[2]^ have primarily emphasized the hepatic aspects of Wilson disease. However, recent literature increasingly recognizes the diverse clinical presentations of the disease, including neurological symptoms.^[3,4]^ This case adds to the growing body of evidence highlighting the importance of considering Wilson disease in atypical presentations and the effectiveness of chelation therapy in addressing these symptoms.

### 7.4. The rationale for conclusions

- The conclusions drawn from this case are based on a combination of clinical findings, genetic analysis, and patient response to intervention. The genetic confirmation of ATP7B mutations, coupled with the positive response to chelation therapy, strongly supports the diagnosis of Wilson Disease as the cause of the patient’s neurological and psychiatric symptoms.

### 7.5. Main takeaway lessons

- This case report underscores several key takeaway lessons:

1. Wilson disease can present with atypical neurological symptoms, making early recognition and diagnosis crucial.
2. A comprehensive diagnostic approach, including genetic analysis, is essential for confirming the diagnosis and initiating appropriate treatment.
3. Personalized interventions and regular monitoring are critical for managing Wilson disease effectively.
4. Psychiatric manifestations can be a part of the disease spectrum, and comprehensive care should address these symptoms in addition to neurological manifestations.

## 8. Patient perspective

At the onset of my illness, the tremors, dystonia, and slurred speech had taken a toll on my daily life and self-esteem. Initially, I was prescribed antipsychotic medications to address my psychiatric manifestations, which had limited effect. However, following the diagnosis of Wilson disease and the initiation of chelation therapy, I witnessed a remarkable improvement in my neurological symptoms. The tremors and dystonia gradually diminished, allowing me to regain control of my movements. Furthermore, the comprehensive care that addressed not only my physical but also my psychological well-being played a vital role in my recovery. While the journey isn’t without its challenges, the treatments have given me hope and a chance to lead a fulfilling life.

## Author contributions

**Conceptualization:** Chukwuka Elendu, Dependable C. Amaechi, Klein A. Jingwa, Tochi C. Elendu.

**Data curation:** Chukwuka Elendu, Dependable C. Amaechi, Klein A. Jingwa, Tochi C. Elendu.

**Formal analysis:** Chukwuka Elendu, Dependable C. Amaechi, Klein A. Jingwa, Tochi C. Elendu.

**Funding acquisition:** Chukwuka Elendu, Dependable C. Amaechi, Klein A. Jingwa, Tochi C. Elendu.

**Investigation:** Chukwuka Elendu, Dependable C. Amaechi, Klein A. Jingwa, Tochi C. Elendu.

**Methodology:** Chukwuka Elendu, Dependable C. Amaechi, Klein A. Jingwa, Tochi C. Elendu.

**Project administration:** Chukwuka Elendu, Dependable C. Amaechi, Klein A. Jingwa, Tochi C. Elendu.

**Resources:** Chukwuka Elendu, Dependable C. Amaechi, Klein A. Jingwa, Tochi C. Elendu.

**Software:** Chukwuka Elendu, Dependable C. Amaechi, Klein A. Jingwa, Tochi C. Elendu.

**Supervision:** Chukwuka Elendu, Dependable C. Amaechi, Klein A. Jingwa, Tochi C. Elendu.

**Validation:** Chukwuka Elendu, Dependable C. Amaechi, Klein A. Jingwa, Tochi C. Elendu.

**Visualization:** Chukwuka Elendu, Dependable C. Amaechi, Klein A. Jingwa, Tochi C. Elendu.

**Writing – original draft:** Chukwuka Elendu, Dependable C. Amaechi, Klein A. Jingwa, Tochi C. Elendu.

**Writing – review & editing:** Chukwuka Elendu, Dependable C. Amaechi, Klein A. Jingwa, Tochi C. Elendu.
